# EVALUATING WAYFINDING INFRASTRUCTURES FOR PEOPLE LIVING WITH DEMENTIA—THE 360 DEGREE MAPS STUDY DENHB

**DOI:** 10.1093/geroni/igad104.0770

**Published:** 2023-12-21

**Authors:** Janissa Altona, Henrik Wiegelmann, Emily Mena, Julia Misonow, Benjamin Schuez, Karin Wolf-Ostermann

**Affiliations:** University of Bremen, Institute of Public Health and Nursing Research, Bremen, Bremen, Germany; University of Bremen, Bremen, Bremen, Germany; University of Bremen, Bremen, Bremen, Germany; University of Bremen, Bremen, Bremen, Germany; University of Bremen, Bremen, Bremen, Germany; University of Bremen, Bremen, Bremen, Germany

## Abstract

Recent studies point to the importance of the built environment for the cognitive and social health of community-dwelling people living with dementia. One key aspect is wayfinding infrastructure, especially in urban areas. However, currently there are no clear indicators of wayfaring infrastructure and no standardized assessment instruments. Here, we provide such an instrument with the potential for online digital assessment as the result of the German DenHB-study. Based on an umbrella review and further research, we first developed a questionnaire to evaluate local wayfinding structures which are sectioned in different thematic blocks like general streetscape, signage, public transportation, buildings etc. Second, we conducted a cross-sectional validation study in the urban area of Bremen, Germany using Apple Maps Street View (2022) with a 360-degree viewing angle at more than 3,000 randomly selected geo coordinates within urban districts with a high proportion of older residents (>65 years). The results document supportive as well as inhibiting wayfinding structures from the perspective of people living with dementia and can also form a basis for a later automated digital evaluation of wayfinding structures. Hence, providing easy to obtain new insights helps to support the development of actions to promote the dementia-friendliness of urban living structures.

